# The use of workflows in the design and implementation of complex experiments in macromolecular crystallography

**DOI:** 10.1107/S090744491201863X

**Published:** 2012-07-17

**Authors:** Sandor Brockhauser, Olof Svensson, Matthew W. Bowler, Max Nanao, Elspeth Gordon, Ricardo M. F. Leal, Alexander Popov, Matthew Gerring, Andrew A. McCarthy, Andy Gotz

**Affiliations:** a European Molecular Biology Laboratory, 6 Rue Jules Horowitz, BP 181, 38042 Grenoble, France; b Unit of Virus Host-Cell Interactions, UJF–EMBL–CNRS, UMI 3265, 6 Rue Jules Horowitz, 38042 Grenoble CEDEX 9, France; c Structural Biology Group, European Synchrotron Radiation Facility, 6 Rue Jules Horowitz, 38043 Grenoble, France

**Keywords:** workflows, automation, data processing, macromolecular crystallography, experimental protocols, characterization, reorientation, radiation damage

## Abstract

The automation of beam delivery, sample handling and data analysis, together with increasing photon flux, diminishing focal spot size and the appearance of fast-readout detectors on synchrotron beamlines, have changed the way that many macromolecular crystallography experiments are planned and executed. Screening for the best diffracting crystal, or even the best diffracting part of a selected crystal, has been enabled by the development of microfocus beams, precise goniometers and fast-readout detectors that all require rapid feedback from the initial processing of images in order to be effective. All of these advances require the coupling of data feedback to the experimental control system and depend on immediate online data-analysis results during the experiment. To facilitate this, a *Data Analysis WorkBench* (*DAWB*) for the flexible creation of complex automated protocols has been developed. Here, example workflows designed and implemented using *DAWB* are presented for enhanced multi-step crystal characterizations, experiments involving crystal re­orientation with kappa goniometers, crystal-burning experiments for empirically determining the radiation sensitivity of a crystal system and the application of mesh scans to find the best location of a crystal to obtain the highest diffraction quality. Beamline users interact with the prepared workflows through a specific brick within the beamline-control GUI *MXCuBE*.

## Abstract 2

A powerful and easy-to-use workflow environment has been developed at the ESRF for combining experiment control with online data analysis on synchrotron beamlines. This tool provides the possibility of automating complex experiments without the need for expertise in instrumentation control and programming, but rather by accessing defined beamline services.

## 1. Introduction

The advances made in synchrotron-based X-ray diffraction experiments for macromolecular samples have made the collection of routine data accessible to non-expert users. These advances rest heavily on the automation of beam delivery, sample handling and online data analysis (ODA; Beteva *et al.*, 2006 ▶; Cipriani *et al.*, 2006 ▶; Gabadinho *et al.*, 2010 ▶; McPhillips *et al.*, 2002 ▶; Nurizzo *et al.*, 2006 ▶; Ohana *et al.*, 2004 ▶; Popov & Bourenkov, 2003 ▶; Soltis *et al.*, 2008 ▶; Stepanov *et al.*, 2011 ▶). The automation linked to each data collection becomes increasingly complex as a function of the sample quality, the number of samples to be processed and the type of experimental data required, which are all foreseen to increase dramatically in the future. Macromolecular crystallography beamlines have seen huge advances in automation for beamline control and sample manipulation. Coupled with the use of fast detectors, this means that data can be collected more rapidly than ever before; therefore, the need to have real-time feedback from data processing and quality monitoring has become critical.

The routine use of *EDNA* (Incardona *et al.*, 2009 ▶) by the ESRF user community to predict optimized data-collection strategies has highlighted the added value of ODA and feedback prior to data collection. *In operando* automatic data reduction (Winter, 2010 ▶; Holton & Alber, 2004 ▶; Incardona *et al.*, 2009 ▶; Leslie *et al.*, 2002 ▶), now implemented at many synchrotrons, also encourages the increasingly efficient use of available beam time. As a consequence of these advances, complex and sequential data collections become feasible, but these experiments are not yet automated and remain the exception. The design and implementation of a suite of high-throughput sample-evaluation beamlines for macromolecular crystallography (MASSIF) as part of the ESRF Upgrade Program (http://go.esrf.eu/Upgrade) has precipitated the need to automate the more complex data-collection workflows which we envisage to be essential for the success of the advanced screening and data-collection experiments to be undertaken. In particular, complex screening processes (Aishima *et al.*, 2010 ▶; Bowler *et al.*, 2010 ▶; Song *et al.*, 2007 ▶; Hilgart *et al.*, 2011 ▶), although already semi-automatic, require incorporation into the existing beamline environment in a robust and reliable way.

An additional challenge is that many of these workflows will continue to evolve with the beamline technology, so a modular architecture is essential. These requirements include access to experimental parameters such as beamline settings and sample information, as well as channels for feeding processing results back to the control system; for example, the need to collect additional data sets. As synchrotrons and beamlines use different control systems [*e.g.* *spec* (http://www.certif.com/), *TANGO* (http://www.tango-controls.org/), *EPICS* (http://www.aps.anl.gov/epics/), *TINE* (http://adweb.desy.de/mcs/tine/) and *NOMAD* (http://www.ill.eu/instruments-support/instrument-control/software/nomad/)], specific developments are needed to implement them in standard and generic data-processing applications [*e.g.* *MOSFLM* (Leslie, 2006 ▶), *XDS* (Kabsch, 2010 ▶), *DENZO* (Otwinowski & Minor, 1997 ▶), *LABELIT* (Sauter *et al.*, 2004 ▶; Zhang *et al.*, 2006 ▶), *BEST* (Bourenkov & Popov, 2010 ▶; Popov & Bourenkov, 2003 ▶), *DNA* (Leslie *et al.*, 2002 ▶), *RADDOSE* (Paithankar & Garman, 2010 ▶), *EDNA* (Incardona *et al.*, 2009 ▶), *STAC* (Brockhauser *et al.*, 2011 ▶) and *STRATEGY* (Ravelli *et al.*, 1997 ▶)]. Additionally, all settings should be stored and made available to other applications in a database such as the LIMS system *ISPyB* (Delagenière *et al.*, 2011 ▶) that is in operation at the ESRF, MAX-lab and Diamond Light Source. It provides a platform where processing applications can find the required input parameters and store their results for subsequent transfer to beamline-control applications (Gabadinho *et al.*, 2010 ▶; McPhillips *et al.*, 2002 ▶; Stepanov *et al.*, 2011 ▶).

Although some pipeline experiments have been developed and are available at the ESRF (Beteva *et al.*, 2006 ▶), the need to implement more complex workflows poses a number of challenges, as many procedures require manual optimization of beamline components. Specific sequences are automated, and software exists, but the integration of software pipelines involves extensive communication between different modules, such as *EDNA*, *ISPyB* and *MXCuBE*, as well as input from the users themselves.

In order to facilitate the development of these protocols and to integrate them quickly into the beamlines, we have designed an intuitive graphical user interface (GUI) called the *Data Analysis Work Bench* (*DAWB*). The workbench provides a framework and structure on which beamline scientists build complex process workflows; these are then provided in a transparent manner to beamline users through the standard beamline-control GUI *MXCuBE* at the ESRF. This paper describes our solution for implementing ODA by connecting data processing with beamline control in a flexible environment. The abstraction of operations into workflow modules has helped in separating instrument control from experimental protocol pipelining, which can result in more robust instrument services and faster development of novel protocols. Some workflows for answering different scientific questions, and how they have been implemented using a workbench tool, are described. The automation of these protocols and their deployment in a user-friendly manner makes complex and laborious experiments possible even for non-expert beamline users. Changing the timescale of these experiments increases efficiency and new science will undoubtedly be enabled.

## 2. Experimental methods and results

### 2.1. Workflows

**Figure 1:**
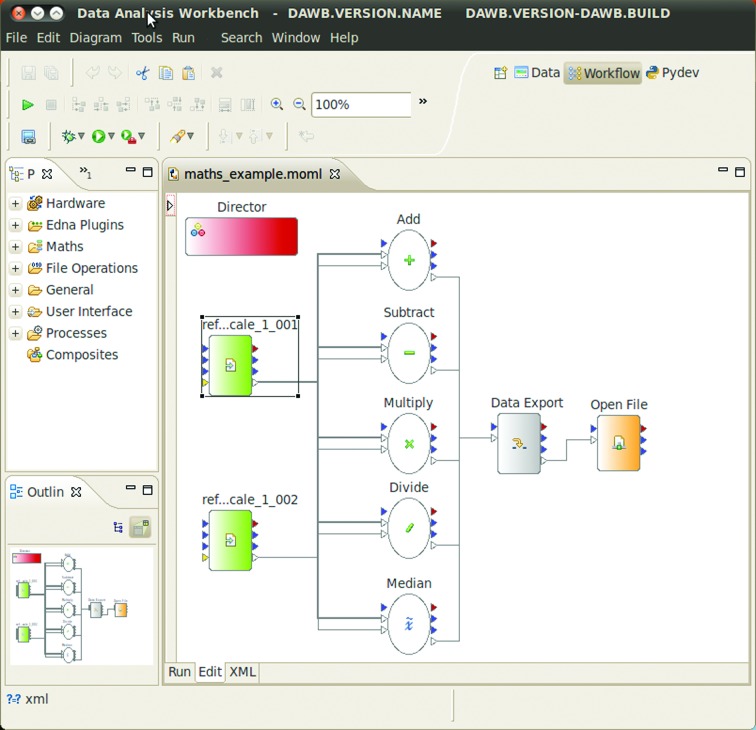
The GUI for designing workflows, as embedded in *DAWB*. The Palette View on the left organizes the available actors into groups such as Hardware/*EDNA*/UI and makes them available to drag and drop onto the main canvas. The workflow shown performs different image-manipulation tasks concurrently and stores the generated results in an hdf5 file which is then opened to visualize the results.

Workflows (sometimes called pipelines) are chains of actions or ‘actors’ (Taylor *et al.*, 2007 ▶) linked by an underlying communication protocol that ensures that the actors are able to correctly interact with each other. In a typical workflow, messages are passed between actors, which generally execute a particular algorithm. However, multiple messages may also exist in a workflow at any one time. In such cases the actors are composed of ‘sources’, which can add jobs to the pipeline queue, ‘transformers’, which complete tasks within the workflow, and ‘sinks’, which receive and stop the propagation of the current message flow. In general, workflows have many advantages over textual languages for data-analysis tasks. When implemented well, workflows standardize the way multiple analysis programs are called by providing a single interface, use a standard data model for communication and contain a graphical editor that allows scientists to interact and can run in a nongraphical mode as a server. Here, a set of sequential and/or parallel actions are defined using actors. The underlying links between the actors can then be handled by a workflow engine. The use of such engines allows an efficient user model to be developed, as it enables CPU multi-threading. Therefore, when a user creates a complex workflow with many branches and/or multiple jobs it avoids the need for the creator to program such threads directly. A GUI greatly enhances workflow engines by allowing the graphical design of workflows (Fig. 1 ▶) with additional features, such as cheat sheets, which provide direct and intuitive access to interactive documentation. These features have a variety of applications in the collection and processing of macromolecular crystallo­graphic data; therefore, a workflow engine was thought to be worth further investigation. Such an engine can enhance the efficient development, testing and rapid deployment of scientific algorithms for the macromolecular crystallography community; for example, the use of crystal realignment or burning strategies, which both incorporate data collection and data analysis in a single visually editable algorithm. One of the most important features is the ability to view the model with a graphical interface as a description or documentation for easy interpretation.

### 2.2. The workflow tool

A Ptolemy II-based engine (http://ptolemy.berkeley.edu/ptolemyII/) with an Eclipse/RCP graphical interface (RCP, the Rich Client Platform, provides robust support for complex GUI construction with multiple features that can be easily incorporated; http://www.eclipse.org) that could support *EDNA* ‘actors’ was selected as the workflow engine (Eker *et al.*, 2003 ▶). This engine, Passerelle, is in use at SOLEIL in its Swing-based form (Abeillé *et al.*, 2007 ▶) and contains advanced multi-threading capabilities. Enabling RCP integration, an Eclipse/RCP-based version was developed, which was released under an Open Source license. The RCP version is based on the Graphical Editing Framework (GEF; http://www.eclipse.org/gef/). GEF has significant support from the open-source and commercial communities and is seen as the leader owing to its user-interface abilities. It was decided to bundle the workflow engine and the GUI for designing workflows into a product we call the *Data Analysis Workbench* (*DAWB*; http://www.dawb.org) and to release the binaries and source code to the open-source community. The bundling mechanism chosen for the source code developed was OSGI (http://www.osgi.org) and the Eclipse platform was used for the workbench GUI. This design allows the code to be bundled and added to the distributed software, enabling custom actors to be developed and integrated into the workbench. The workflow-execution process and the workbench graphical interface are separate processes. They communicate using a configurable layer based on Java messaging extensions (JMX; http://www.oracle.com/technetwork/java/javase/tech/javamanagement-140525.html). It was decided to enable the Ptolemy II composite design, allowing sub­models to be created and reused in different workflows. This design also helps in generalizing actions, such as data collection, and hiding differences in specific solutions, such as the interface between varying beamline-control systems at different synchrotrons. Separating beamline control from experimental sequences also helps in the robust implementation of the required beamline services by clearly defining and reducing the scope and development time. This allows the designed workflows to be easily shared between synchrotron-radiation facilities.

### 2.3. Experimental applications

**Figure 2:**
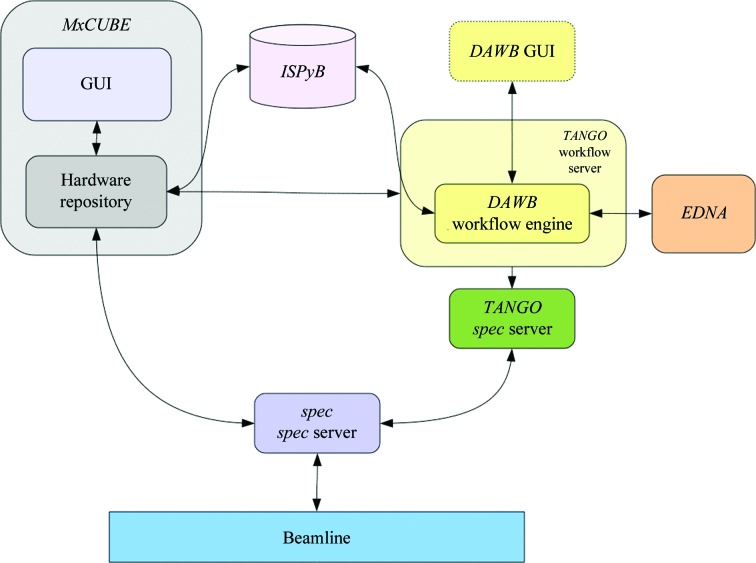
Diagram showing the integration of workflows at the ESRF MX beamlines. Arrows indicate information exchange between software and hardware components.

The integration of workflows into the ESRF beamline experimental environment is a two-step process. Firstly, the workflow is prototyped using *DAWB*, which provides an easy-to-use graphical tool for connecting and configuring processes (actors). Once a workflow has been prototyped and tested, it is deployed and made available in *MXCuBE* in a newly developed workflow brick which communicates with the workflow engine *via* a *TANGO* workflow server. Interaction with the beamline user is handled by *MXCuBE*. Although the Passerelle engine can take emergency action or send signals to trigger protocols for certain cases (such as hardware failure; as discussed by, for example, Abeillé *et al.*, 2007 ▶), they are not used in the currently available workflows as the required instrument-control services have not yet been implemented. Currently, beamline users can launch, pause or abort the workflows from the *MXCuBE* GUI. The workflow engine used is the same in both these cases, but the *DAWB* GUI is used to construct the workflow, while *MXCuBE* is used for beamline–user inter­action and control. Designing and executing workflows on the fly, as provided by the *DAWB* GUI, is an efficient method for experiment design which is of interest to all scientists and not just to beamline staff. A proper beamline-service layer that offers automatic status checks, interlock systems for avoiding hardware problems, such as collisions during instrument movements, as well as intuitive problem-resolution strategies could allow this option to be enabled for all users, but is not yet available to them. However, the current system requires special care and attention during the design phase, leaving the responsibility of preparing the workflows in the hands of beamline scientists. Currently, the workflow engine and *MXCuBE* access the beamline hardware together through *spec*. We have developed a separate *TANGO* server on top of the *spec* client to standardize the beamline hardware access from the workflow actors. This model can help in the specification of a beamline service layer at the ESRF. Fig. 2 ▶ shows an overview of the implementation of experiment control from *MXCuBE* and the workflow engine.

Here, we describe the implementation of several MX experiment types that are complex and difficult to fully automate using other methods. All sub-workflows hidden in the composite actors used in the following examples are available in the Supplementary Material^1^.

#### 2.3.1. Enhanced *EDNA* crystal characterization workflow

**Figure 3:**
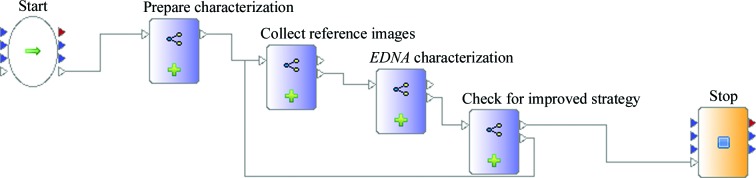
A workflow for Enhanced Characterization. By coupling subsequent and related steps into composite actors, such as those coloured blue, the higher level logical sequence of the experimental protocol is preserved and clearly presented to non-programmers.

Probably the best-known automation of ODA for MX beamlines is sample characterization. Both *DNA* (Leslie *et al.*, 2002 ▶), and more recently *EDNA* (Incardona *et al.*, 2009 ▶), had the initial goal of automating the sequence of taking reference images, characterizing these images and calculating an optimized data-collection strategy, taking into account user requirements and radiation damage. At the ESRF, the automation of such characterization has been available to users since 2005 (Beteva *et al.*, 2006 ▶). The shortcoming of the current implementation is that if reference images are not optimally collected (for example, using an incorrect exposure time, oscillation width and/or detector resolution) it is difficult, if not impossible, to calculate the optimal data-collection strategy. *EDNA*/*BEST* can give advice on the optimal detector resolution (Popov & Bourenkov, 2003 ▶); however, the user has to manually follow suggested values and restart the collect and characterize pipeline. The complexity of such a workflow, in which ODA results are fed back to instrument control and result in a new hardware action, exceeds the capabilities of the experiment-control software. Using *DAWB*, we have enhanced the characterization pipeline by simply adding the optional step of automatically re-collecting reference images with a different exposure time or oscillation value and detector resolution (Fig. 3 ▶). If the workflow is run in interactive mode the user can easily intervene and interrupt the workflow at certain stages as well as change the proposed new data-collection parameters through prompts displayed in *MXCuBE*, if needed. Using the workflow tool, the design of a new experimental protocol or an extension of an existing one, such as the case of Enhanced Characterization, becomes simple as all the required tools for ODA and beamline actions are available and can be combined. Such a natural enhancement of the characterization pipeline has long been in demand but has not been implemented. Using *DAWB*, its creation and deployment took only a single day.

#### 2.3.2. Crystal radiation-sensitivity measurement workflow

**Figure 4:**
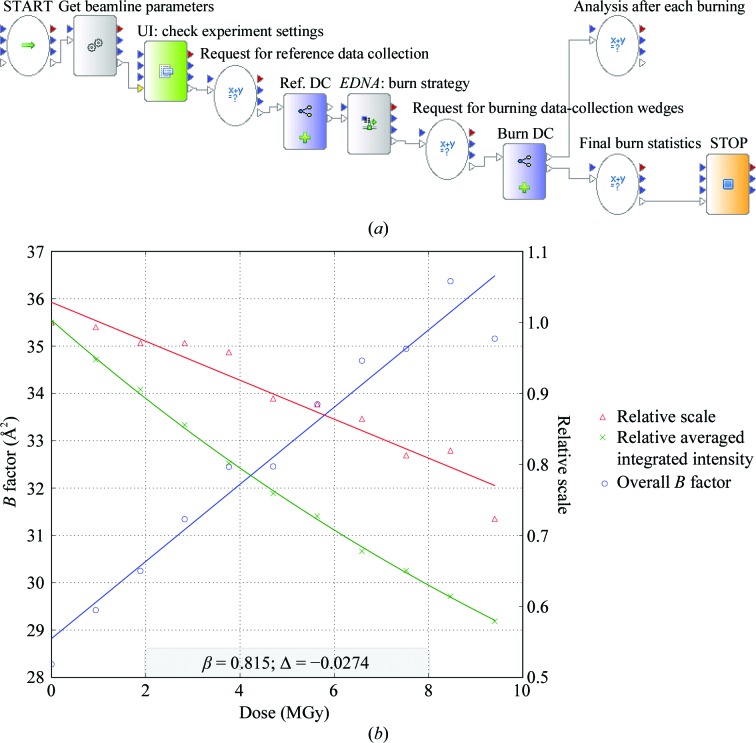
(*a*) The radiation-sensitivity workflow and (*b*) its output plot as presented by *DAWB* for a cubic insulin crystal. Markers show measured values, whilst fitting curves are denoted by solid lines. Overall *B* factors are represented in blue, relative scales in red and the relative averaged integrated intensities are shown in green. Linear fitting curves are applied to both overall *B* factors and relative scales, whereas the relative averaged integrated intensities are fitted with an exponential curve. The horizontal axis shows the calculated dose (*RADDOSE*). The radiation-damage sensitivity coefficient (β = 0.8 Å^2^ MGy^−1^) and the slope of the relative scale fitting line (Δ = −0.027 MGy^−1^) are shown at the bottom of the plot.

The radiation damage that occurs during data collection in MX limits the information that can be obtained from a single crystal (see, for example, Garman, 2010 ▶; Krojer & Delft, 2011 ▶). Therefore, consideration of radiation-damage effects is critical for optimal data-collection planning. Most radiation-damage phenomena are proportional to the absorbed dose and can be accurately predicted if the experimental conditions are well known. Routine measurement of the X-ray beam size, profile and flux, together with knowledge of the chemical composition of the sample, are of great importance for calculating the absorbed dose using *RADDOSE* (Paithankar & Garman, 2010 ▶). When the sample sensitivity or the beam-flux calibration is uncertain, a reliable experimental protocol is necessary to empirically calibrate a linear damage model. This procedure requires the sacrifice of a whole crystal or part of a crystal. It involves measuring the degree of damage in a sample, or part of it, by repeated exposure of the crystal to X-rays (Leal *et al.*, 2011 ▶). Such a protocol to determine the radiation sensitivity of a crystal has been established and implemented in *DAWB*. After an initial reference data collection, *EDNA* provides a crystal-burning strategy plan consisting of 11 successive collections of the same 3° wedge of data (the collecting cycle) interleaved with longer X-ray exposures to burn the crystal (the burning cycle). The workflow then initiates the immediate analysis of the images collected after each step (Fig. 4 ▶ *a*). The computational implementation burden of consecutive requests to the beamline instrumentation devices and to processing crystallography software calls in parallel is lightened by the ease of using a workflow. The radiation-sensitivity information extracted from this protocol can then be directly used for the optimal planning of a data-collection strategy which takes into account the predicted radiation-damage-induced decay in diffraction intensities. Gathering this workflow into a composite actor, the procedure can be reused in* DAWB* for further enhancing crystal characterization by making an initial step of sacrificing a part of the crystal to apply a correct crystal-decay model during the data-collection strategy calculation at a different location. Using test crystals with well known radiation sensitivity, the procedure can also be used at the beamline to verify and calibrate the flux and beam size (Leal *et al.*, 2011 ▶).

To test the procedure through *DAWB* under real conditions, a crystal of cubic insulin belonging to space group *I*2_1_3, with unit-cell parameters *a* = *b* = *c* = 77.93 Å, obtained as described by Nanao *et al.* (2005 ▶) was used. The measurements were carried out on ESRF beamline ID14-4 (McCarthy *et al.*, 2009 ▶), where an ADSC Q315 detector is installed. The beam size was defined by two slits and set to 100 µm vertically and 100 µm horizontally at the sample position. The incident monochromatic beam with an energy of 13.2 keV had a flux of 2.7 × 10^12^ photons s^−1^. The procedure for dose calculations was applied without specifying the exact chemical composition of the sample, *i.e.* assuming the *EDNA* default composition for an average protein crystal (47% solvent, 0.05 S atoms per amino-acid residue and 300 m*M* sulfate in the buffer solution). The coefficient obtained (β = 0.8 Å^2^ MGy^−1^) can be used for flux and beam-size calibration (Fig. 4 ▶ *b*). According to Kmetko *et al.* (2006 ▶), all protein crystals may be comparably radiation sensitive at 100 K, with a constant coefficient of sensitivity to absorbed dose (within a factor of two) of approximately β ≃ 1 Å^2^ MGy^−1^. The value obtained is thus consistent with the observation of Kmetko *et al.* (2006 ▶) and supports the utilization of this procedure for validating beamline calibration. As a composite actor, this protocol can be easily reused in other workflows such as a verification step after beamline calibration or as a routine item in automatic beamline-testing procedures.

#### 2.3.3. Kappa-reorientation workflow

**Figure 5:**
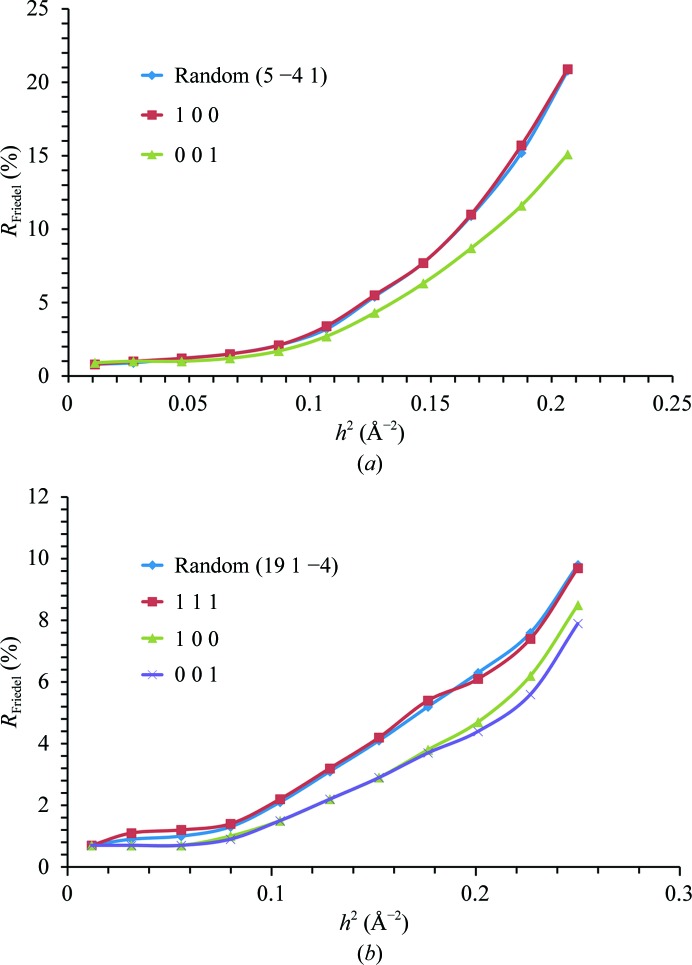
Simulation of the crystal orientation effect on achievable minimum noise between Bijvoet mates represented as *R*_Friedel_ = 〈|〈*E* ^2+^〉 − 〈*E* ^2−^〉|〉, where 〈*E* ^2+^〉 and 〈*E* ^2−^〉 are normalized average intensities of Bijvoet mates plotted as a function of resolution. Calculations were performed by the program *BEST* accounting for radiation-damage effects in the cases of (*a*) trypsin, space group *P*3_1_21, and (*b*) thaumatin, space group *P*4_1_2_1_2.

The use of kappa goniometers for crystal reorientation can be favourable in different scenarios in MX (Brockhauser *et al.*, 2011 ▶). These include the case where Bijvoet pairs of reflections (a reflection and the Friedel pair of its symmetry equivalent, *e.g. hkl* and 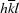), can be measured on the same diffraction image by properly aligning an even-fold symmetry axis along the spindle. Hence, anomalous differences can be measured at the same time and radiation-damage-induced non-isomorphism (Blake *et al.*, 1962 ▶; Hendrickson, 1991 ▶) within these Bijvoet pairs can be minimized, resulting in more accurate measurements of the anomalous differences. Aligning a specific symmetry axis can result in the collection of a complete data set within a reduced rotation range (Dauter, 1999 ▶) so that the total dose can be lower, leading to less severe radiation damage. Fig. 5 ▶ shows the advantage of aligning symmetry axes. Comparative simulations (based on experimental data from ESRF beamline ID23-1) have been carried out using the program *BEST* (Bourenkov & Popov, 2010 ▶) for trypsin (*P*3_1_21) and thaumatin (*P*4_1_2_1_2) to show how the noise in the anomalous signal can be reduced. Another example of an advantageous crystal reorientation is the alignment of the densest axis in reciprocal space, usually corresponding to the longest unit-cell axis. By aligning this axis parallel to the spindle, the overlap of spots can be minimized (Dauter, 1999 ▶).

**Figure 6:**
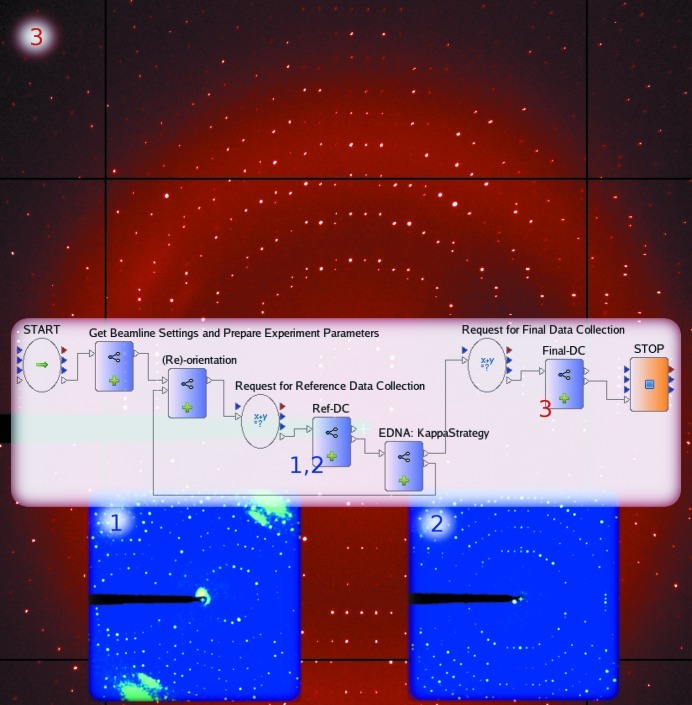
The kappa-reorientation workflow with example diffraction images captured from the same FAE crystal in different orientations. The blue image on the left is taken at step 1 in the initial random orientation. The blue image on the right is the reference image at step 2 in an aligned orientation to optimize the strategy for a complete data collection in this orientation. The red background image is taken at step 3 as the first image of the final data set.

Precise kappa goniometers and properly calibrated inverse kappa goniometers (Brockhauser *et al.*, 2011 ▶), such as the EMBL/ESRF MiniKappa, support sample reorientation while retaining the centred position of the crystal, which allows their use as a pure rotational goniometer (Paciorek *et al.*, 1999 ▶). After determining the initial orientation of the sample, which involves the measurement and indexing of diffraction images, a set of preferred orientations and the required changes in goniometer settings can be computed. Using *STAC* (McCarthy *et al.*, 2009 ▶) with *MOSFLM* or *XDS* on the ESRF MX beamlines, such a procedure can be carried out manually (http://go.esrf.eu/MiniK). Data-processing tools, such as *EDNA* or *RAPD* (Kourinov *et al.*, 2011 ▶), allow the automated calculation of reorientations, but the process involves the use of several different software packages and beamline GUIs. The International Kappa Workgroup (http://www.epn-campus.eu/kappa/) has defined a protocol for automating the use of kappa goniometers. This is a three-step iterative protocol which consists of initial characterization of the sample, the calculation of a set of preferred orientations and testing the diffraction quality and predicting data-collection statistics at different orientations until a satisfactory result is achieved. Although *EDNA* plugins for the reorientation calculations were prepared in 2009, an integrated pipeline could not be built and made available on the beamlines as the control-system implementation is very complex and even small changes can result in unforeseen problems. In such an environment, a large amount of testing is required for even a small modification. Within a week (from which a single day was allocated on the beamline) using the Workflow Tool in *DAWB* the required data-analysis tools implemented in *EDNA* were combined with experimental control actors for beamline preparation and goniometer setup, a GUI for initiating data collections, collecting reference images and full data sets and activating the sample-viewing camera to monitor the samples during reorientations. A snapshot of the implementation of this workflow is shown in Fig. 6 ▶. Using this workflow, the sample is automatically characterized, the beamline and detector settings together with the goniometer settings are optimized and the suggested full data sets are collected. The implemented workflow provides a menu allowing the selection of re-orientation targets defined in *EDNA*. These allow users to choose the most appropriate strategy according to the needs of their samples. ‘Smallest Overall Oscillation’ allows the reorientation of the crystal, so the collection of a single-sweep complete data set would require the minimum overall oscillation. The ‘Cell’ option aligns a reciprocal unit-cell axis along the spindle. This option is useful for verifying crystal symmetries as well as investigating spot overlaps. The option ‘Anomalous’ aligns an even-fold symmetry axis along the spindle to measure the anomalous signal between the Bijvoet pairs on the same images. Finally, the ‘Smart Spot Separation’ option maximizes spot separation while maintaining the highest possible completeness by avoiding the blind zones during data collection. This is achieved by a slight misalignment of the longest unit-cell edge of the crystal when approaching the optimal orientation.

The user interfaces are defined at different steps in the workflow for verifying sample positions and goniometer settings as well as reviewing suggested beamline settings and data-collection parameters (see Supplementary Material^1^ for user-interface actors; highlighted in green). In the case of ‘automatic mode’ the workflow engine skips these predefined steps of user interactions and runs the whole protocol autonomously. In the following example, a long rod-shaped (150 × 150 × 400 µm) crystal of feruloyl acid esterase (FAE; Davies *et al.*, 2001 ▶; space group *P*2_1_2_1_2_1_; unit-cell parameters *a* = 65.72, *b* = 108.94, *c* = 113.59 Å, α = 90, β = 90, γ = 90°; mosaicity of 0.4°) was exposed at a wavelength of 1.2536 Å. After initial reference-image collection (see Fig. 6 ▶, step 1) at goniometer angles of zero, a reorientation (ω = 224.9°, κ = 75.2°, ϕ = 200.3°) was suggested which aligned a* along the spindle and b* along the beam according to a ‘Cell’ request. Fig. 6 ▶ (step 2) shows the diffraction pattern collected in this orientation where c* is along the beam. After the successful re-characterization, the suggested data-collection strategy in this aligned orientation was composed of a single wedge of 83.3° starting at ω = 139°, allowing the collection of a 96% complete data set at 1.7 Å resolution. After reviewing the suggested strategy, the final data collection was performed 180° away starting at ω = 319° (Fig. 6 ▶, background image) in order to avoid the self-shadow, which could have appeared at high resolution when the MiniKappa enters the diffraction cone. By simply starting the data collection 180° away, the kappa arm does not move between the sample and the detector. A physical model of the goniometer setup allows the automatic detection of such collision or shadow problems and can be added to enable the fully automatic use of the kappa workflow, even in the case of very high-resolution experiments.

**Figure 7:**
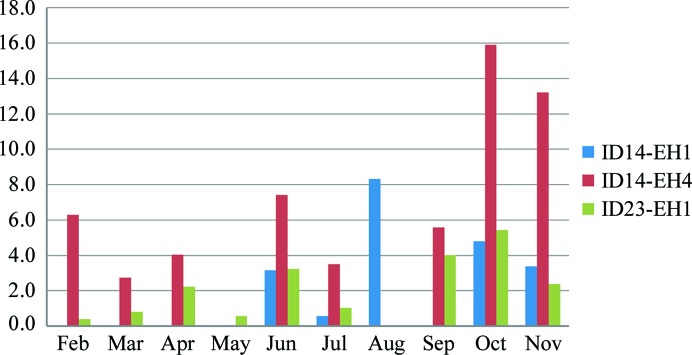
The ratios of data collections using the MiniKappa are shown as a function of time for scheduled beamtime in 2011 on ESRF public MX beamlines. Note that ID23-2 does not have the MiniKappa mounted routinely and MiniKappa usage on ID29 is not available in the beamline operation database.

The deployment of crystal reorientation within *MXCuBE*, the standard beamline-control GUI, its availability through a pull-down menu of options and its guiding interaction steps move these experiments from use only in desperate cases to becoming a standard data-collection protocol. This workflow was deployed on beamline ID14-4 in September 2011. Fig. 7 ▶ shows the doubling of the use of MiniKappa on ID14-4 in October and November, when the workflow was made available *via* *MXCuBE*. Its use on ID23-1 and ID14-1, where the kappa workflow was not offered, remained low. The advantages of reorientation are well documented and its routine use will allow all users to benefit without the need for expert assistance.

#### 2.3.4. Mesh-scan workflow

**Figure 8:**
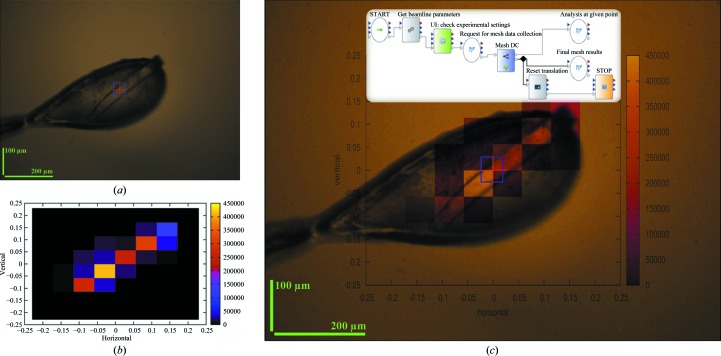
The mesh-scan workflow was executed on a precentered trypsin crystal. (*a*) The on-axis microscope view of the crystal. (*b*) The result of scanning a ±225 µm region of interest both horizontally and vertically with a 50 µm square beam using 57 µm steps is shown as displayed in *DAWB*. (*c*) Overlay of the scan results on the microscope view together with the workflow that was used to perform the experiment.

The large multi-component complexes and membrane proteins now routinely studied in structural biology tend to produce either very small crystals or crystals that can be extremely heterogeneous in their diffraction properties. The increasing availability of microfocused X-ray beams coupled with experimental environments optimized for MX has allowed the design of advanced sample-evaluation (Aishima *et al.*, 2010 ▶; Bowler *et al.*, 2010 ▶; Hilgart *et al.*, 2011 ▶; Song *et al.*, 2007 ▶) and data-collection (Flot *et al.*, 2010 ▶; Hilgart *et al.*, 2011 ▶) protocols. In order to locate very small crystals or the optimum region of a crystal larger than the X-ray beam, mesh scans have been developed to collect an image at numerous points specified within a grid. Reusing the beamline actors prepared for the other workflows presented above, and also integrating ODA applications, the necessary workflow was developed, tested and deployed in *MXCuBE* in 6 h, which is incomparably faster than it would have taken using the traditional tools. The workflow for a simple two-dimensional mesh scan includes actors for beamline preparation and goniometer setup, a GUI for specifying mesh-scan parameters, collection of images from grid points, analysis of data from each point and on-the-fly graphical representation of the data (Fig. 8 ▶). Using this workflow, the entire projection of a crystal can be characterized in terms of its diffraction quality and the results presented to the user automatically in an intuitive manner.

A uniform two-dimensional mesh scan of 57 µm steps was performed on a 380 µm long and 40 µm wide rod-shaped trypsin crystal with a single 1 s exposure at each point using an oscillation of 1°. The images were immediately analysed by *LABELIT* and the results were plotted in a two-dimensional coordinate system aligned with the sample-viewing on-axis microscope with an origin at the actual centred point. The *EDNA* data model was used to define the complete data-collection plan in which, for each wedge to be collected, a separate three-dimensional vector called ‘Sample Position’ is used to describe the positioning of the sample. The two-dimensional screen coordinates, which are the positions on the on-axis viewing system, are calculated for each Sample Position as a function of sample orientation and microscope settings, such as zoom level. Individual diffraction images were immediately processed using distl.thin_client (Adams *et al.*, 2010 ▶) and distl.mp_spotfinder_server_read_file. The total integrated signal metric was used to assemble a two-dimensional plot of diffraction quality as a function of screen coordinates in *GnuPlot* 4.4 and *PM*3*D*. This image (Fig. 8 ▶ *b*) was scaled and superimposed on a screen capture (Fig. 8 ▶ *a*) from *MXCuBE* (Fig. 8 ▶ *c*). Using this workflow, the crystal was quickly found. The workflow can also help in locating the best diffracting crystal volumes in a given projection. Its repetitive application as a subworkflow at different orientations can also be used to automatically perform diffraction-based centring or tomography.

## 3. Discussion

The use of *DAWB* to build enhanced data workflows has led to the fast implementation of several highly complex experiments that offer completely automated execution and are presented to the users through a standard beamline-control GUI. The *DAWB* GUI provides a framework with which programmers and beamline scientists can build workflows together, without the scientist having to become an expert in programming complex and intricate control software or the programmers having to become experts in the experimental protocols. It gives more time to the control-system programmers to focus on providing robust services which can be then used flexibly. This separation requires a clear definition of beamline services. A lesson learned through the development of these workflows is that the creation of a clear beamline-service layer interface is essential. The implementation presented here is now used as the basis of current developments at the ESRF. During a long shutdown (December 2011–May 2012) *MXCuBE* will go through a major refactoring. The changes relating to ODA integration are now following the workflow model. In the future, it is envisaged that beamline scientists with no programming skills will be able to use *DAWB* to design complete workflows without assistance from software developers. These workflows can then be programmed by the software developers and tested in simulation mode to refine data flow and user input prior to testing them on the beamline. Once validated offline, they can be tested in the experimental environment. Finally, the user-control panel can be integrated into the beamline-control GUI and tested prior to being released to all beamline users. The kappa workflow was designed in a week and its deployment and testing on the beamline required only a single day. Reusing its basic components, the subsequent workflows described here required only a day each to implement. This process minimizes the beamtime required for development and avoids potential disruptions introduced by changing the beamline-control GUI. Experiences gained from working with *DAWB* show the importance of using a data model. Such a model enables the abstraction needed for the communication between actors and the construction of composite actors essential for effective unit testing. To guarantee the exportability of the workflows, hardcoded elements should also be minimized and a unique interface defined for all hardware services.

We have demonstrated the use of a workflow tool in the design and automation of several complicated experimental protocols on a synchrotron beamline. The workflow tool is currently being applied to many other experiments, such as controlled crystal dehydration (Russi *et al.*, 2011 ▶; Sanchez-Weatherby *et al.*, 2009 ▶) and diffraction-based auto-centring of crystals (Song *et al.*, 2007 ▶). However, the tool has potential for much greater integration of all experiments in structural biology. The easy linking of modules means that data collection can be directly linked to autoprocessing software and more sophisticated downstream processing of data can also be added, such as *Auto-Rickshaw* (Panjikar *et al.*, 2005 ▶) and *BALBES* (Long *et al.*, 2008 ▶), to solve structures where possible. The tool may also be used to link results from different techniques. For example, SAXS data-collection and reduction workflows could be connected to crystallographic experiments to validate models or provide templates for more advanced modelling protocols. It also provides the possibility of automating processes that are usually upstream of the typical MX experiment. Advances in high-throughput crystallization screening have increased the number of potential protein crystals and the need for *in situ* screening (Jacquamet *et al.*, 2004 ▶). With the possibility of automated mounting of crystals becoming a reality (Berger *et al.*, 2010 ▶; Kitago *et al.*, 2010 ▶; Viola *et al.*, 2007 ▶), workflows that would allow selection of the best protein crystals followed by harvesting and data collection can be imagined.

The use of workflows is radically different to working with a traditional MX experiment-control GUI, in which ‘data collection’ is a single action which finishes after its execution. The workflow server, and its generic integration into *MXCuBE*, enriches the traditional GUI with the provision of new complex workflow possibilities but keeping the general experiment-control interface unchanged. This allows the design, implementation and dissemination of complex experimental protocols that are transparent and intuitive to the user community. The implementation of *DAWB* should allow new protocols to be developed quickly and easily in response to the growing demands of the most challenging projects in structural biology.

## Supplementary Material

Supplementary material file. DOI: 10.1107/S090744491201863X/gm5021sup1.pdf

## Footnotes

1 Supplementary material has been deposited in the IUCr electronic archive (Reference: GM5021). Services for accessing this material are described at the back of the journal.
