# Supplementary figures and images for: The use of workflows in the design and implementation of complex experiments in macromolecular crystallography
Source: Acta Crystallogr D Biol Crystallogr. 2012 Jul 17;68(Pt 8):975–84. doi: 10.1107/S090744491201863X (PMC3413211; doi:10.1107/S090744491201863X)

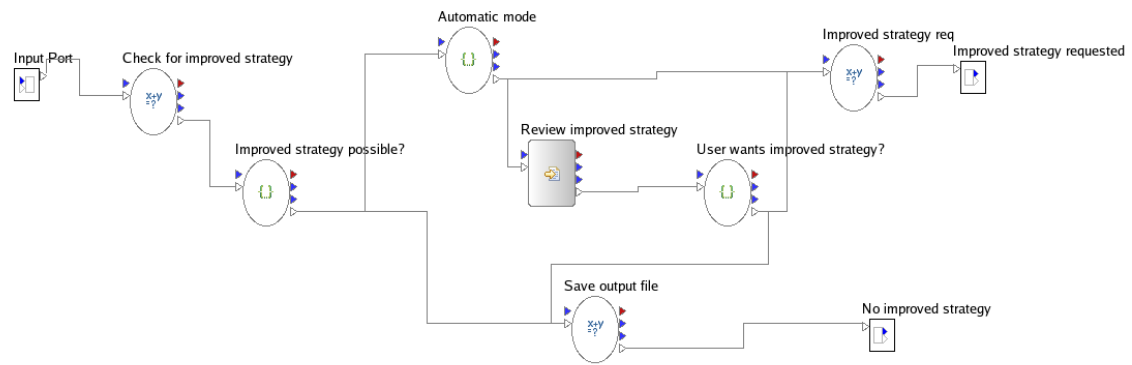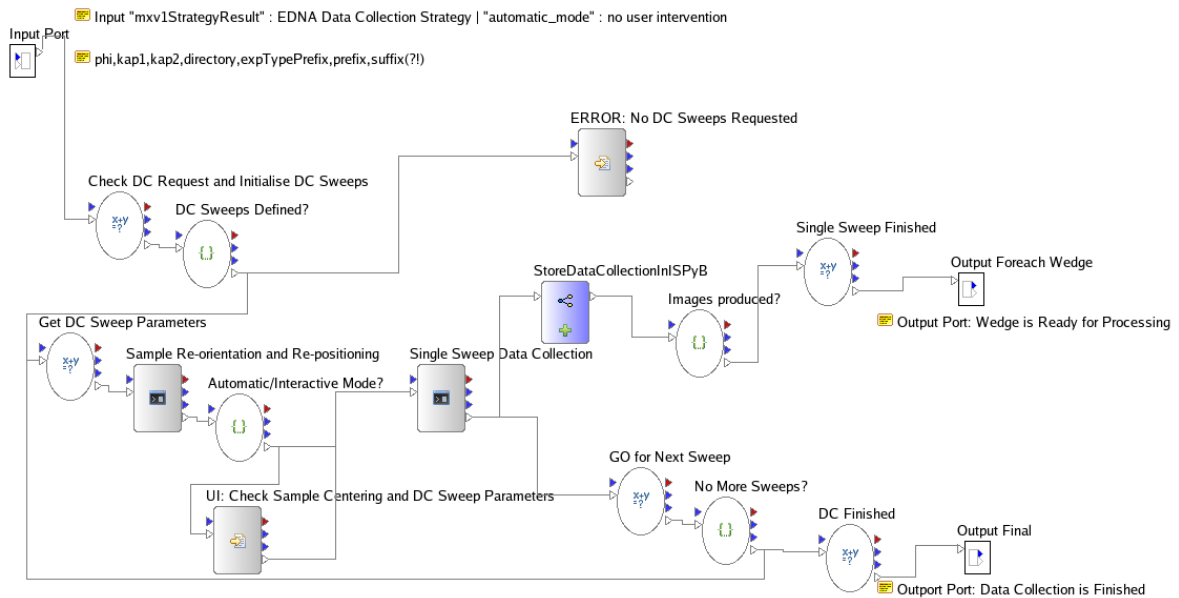

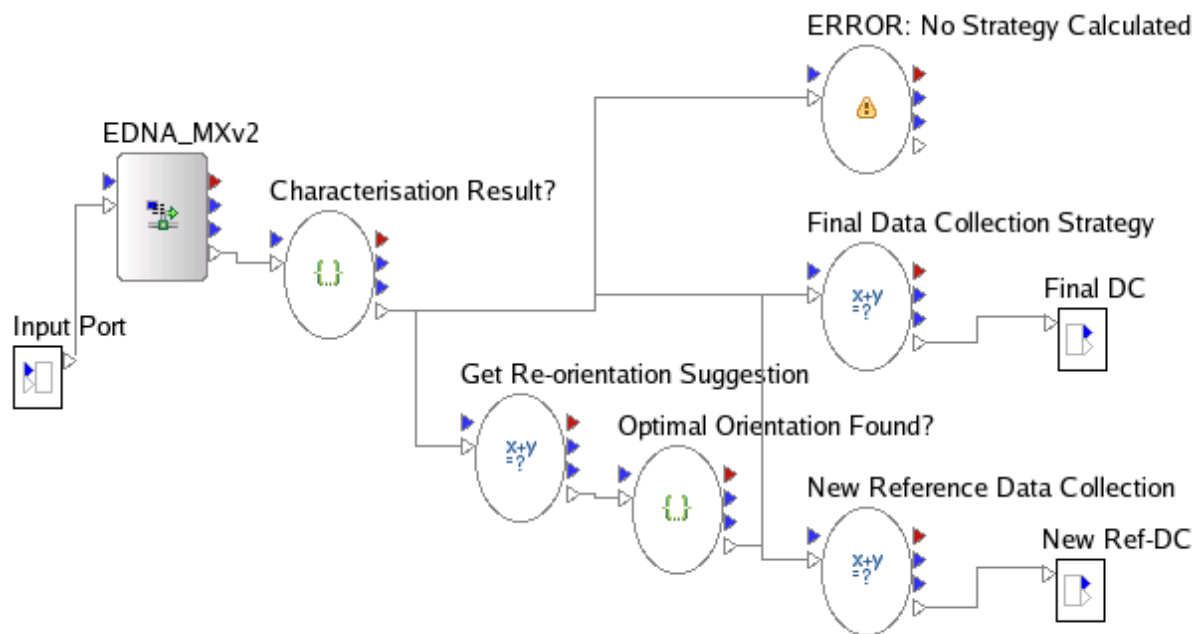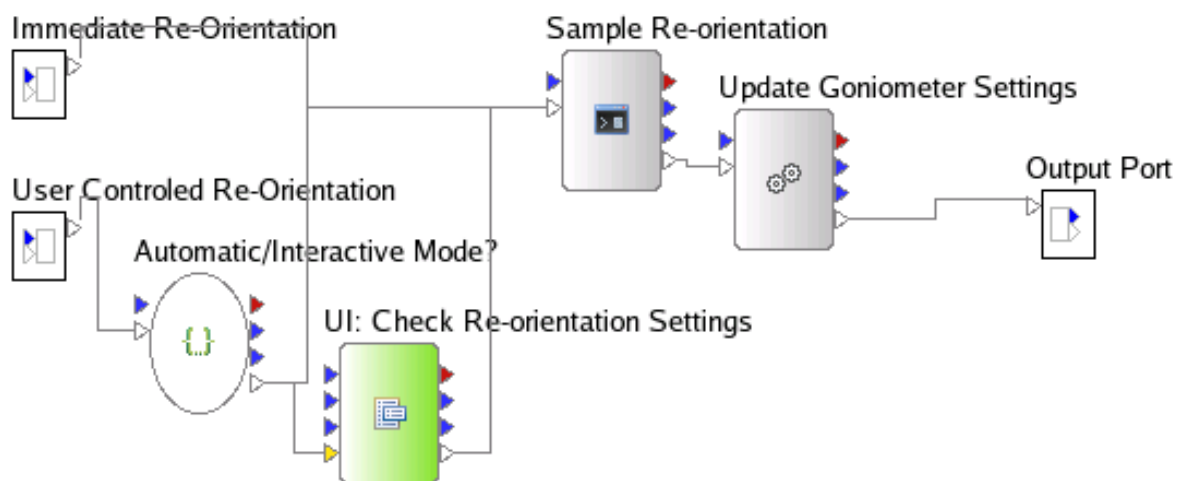

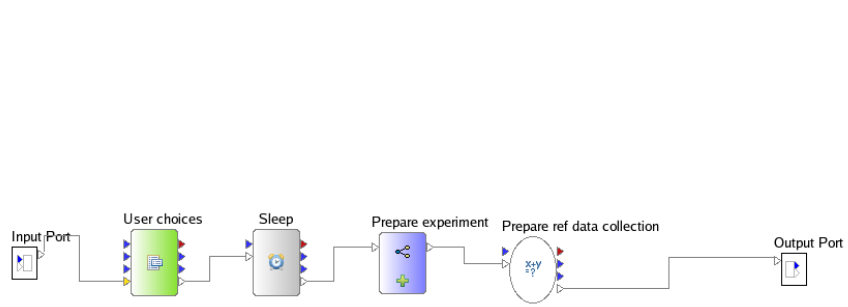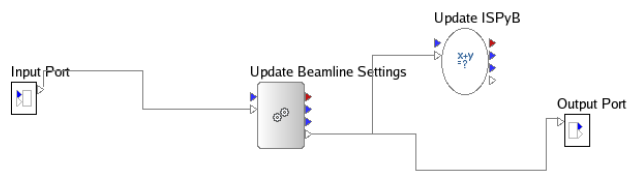

Supplement: Supplementary file 1 [file d-68-00975-sup1.pdf]
